# The role of worry in exercise and physical activity behavior of people with multiple sclerosis

**DOI:** 10.1080/21642850.2022.2112197

**Published:** 2022-08-18

**Authors:** Megan Ware, Patrick O’Connor, Kristen Bub, Deborah Backus, Kevin McCully

**Affiliations:** a Department of Epidemiology and Cancer Control, St. Jude Children’s Research Hospital, Memphis, TN, USA; b Department of Kinesiology, College of Education, University of Georgia, Athens, GA, USA; c Department of Educational Psychology, College of Education, University of Georgia Athens, GA, USA; d Shepherd Center, Atlanta, GA, USA

**Keywords:** Worry, fatigue, physical activity, exercise, multiple sclerosis, mixed methods

## Abstract

**Purpose:**

This study is a secondary analysis of data from a mixed methods exploration of fatigue in people with multiple sclerosis (MS), a chronic autoimmune disease affecting the central nervous system. During initial analysis, worry emerged during discussions of the fatigue experience. The purpose of this study is to explore worry in relationship to exercise and physical activity behavior.

**Methods:**

Mixed methods were used to address the research question. 55 people with MS provided demographic and survey data (habitual physical activity, body perception, functioning). 35 participated in semi-structured interviews on the topics of fatigue, body sensations, emotions, and their effects on physical activity. Qualitative data were analyzed utilizing constructivist grounded theory. Quantitative data were analyzed utilizing multiple regression.

**Results:**

Qualitative theory described participants’ thoughts and experiences regarding the consequences of fatigue during activity, and how they appear to influence subsequent actions. Worry played a critical role in thought processes regarding physical activity. Aspects of body perception were weak quantitative predictors of physical activity behavior after control of physical functionality.

**Conclusions:**

The most significant finding of this study was the description and dialogue about worry as a factor that shapes perceptions of the benefits and value of exercise and physical activity. Physical activity practitioners could benefit from seeking to understand physical-activity-related worry when examining physical activity behavior and designing programming.

## Introduction

Multiple sclerosis (MS) is a chronic autoimmune disease affecting the central nervous system, impacting 2 million people globally (Thompson, Baranzini, Geurts, Hemmer, & Ciccarelli, 2018). MS is a severe, unpredictable neurogenerative disease, characterized by inflammatory demyelination. Consistent use of disease-modifying therapy for MS can reduce risk of relapse by 29–68% (McGinley, Goldschmidt, & Rae-Grant, 2021), but the disease can still result in severe psychological and physical impairment. One of the most commonly reported symptoms in people with MS is fatigue (Krupp, 2006). Fatigue is highly associated with decreased quality of life for people with MS (Krupp, Alvarez, LaRocca, & Scheinberg, 1988). For the purposes of this study, fatigue refers to subjective perception of fatigue (Kluger, Krupp, & Enoka, 2013).

Elevated levels of fatigue, trait and state anxiety, depression, and pain are also associated with elevated levels of worry in people with MS (Bruce & Arnett, 2009), all of which can contribute to emotional distress and lower quality of life. It is important to note that while anxiety and worry are related, evidence in people with MS suggests that they are separate constructs (Bruce & Arnett, 2009). Worry has been found to be related to unpredictability of the physical and mental effects of MS (Desborough et al., 2020). In fact, individuals with MS who mistake minor changes in physical symptoms as exacerbations do have elevated levels of emotional distress (Kroencke, Denney, & Lynch, 2001), implicating a major role of one’s interpretation of bodily status in emotional health.

Although the exact cause of fatigue in MS is unknown, reduced interoception has been proposed as a neurocognitive mechanism contributing to fatigue (Campo et al., 2019). Interoceptive awareness is the relationship between interoceptive accuracy (how accurate one is at detecting body sensations or changes in body sensations) and interoceptive sensibility (sensitivity of an individual to body sensations), and is thought of as a metacognition (Garfinkel, Seth, Barrett, Suzuki, & Critchley, 2015). In people with MS, reduced neural activity, connectivity, and mass in areas of the brain responsible for these metacognitions are associated with both interoceptive deficit and increased fatigue (Harrison et al., 2009; Lansley, Mataix-Cols, Grau, Radua, & Sastre-Garriga, 2013; Rocca et al., 2012; Salamone et al., 2018).

Habitual physical activity, and exercise, have been shown to reduce fatigue levels in people with MS (Moss-Morris et al., 2019; Pilutti, Greenlee, Motl, Nickrent, & Petruzzello, 2013). For the purposes of this study, physical activity refers to consistent behavior, while exercise refers to bouts of physical activity. Furthermore, higher levels of physical activity have been shown to be associated with slower disease progression (Dalgas & Stenager, 2012), improved quality of life (Motl & Gosney, 2008), and lower physical activity is associated with increased symptom severity (Motl et al., 2008a; Motl, Snook, & Schapiro, 2008b). Combinations of physical activity or exercise and mindfulness have also been shown to be effective in reducing worry or the constellation of emotions encompassing emotional distress (Burschka, Keune, Oy, Oschmann, & Kuhn, 2014; Kolahkaj & Zargar, 2015; Mills & Allen, 2000). However, many people with MS are not as active as their healthy counterparts (Kinnett-Hopkins, Adamson, Rougeau, & Motl, 2017). One of the reasons people with MS report they are less active is because of their higher levels of fatigue (Kayes, McPherson, Schluter, Taylor, Leete, & Kolt, 2011a; Ploughman, 2017). Although not all people with MS report fatigue as a barrier to physical activity (Vanner, Block, Christodoulou, Horowitz, & Krupp, 2008), when present, fatigue can make it difficult to initiate physical activity, often for the sake of conserving energy for other important daily tasks. This presents an interesting paradox: increased fatigue can lead to less physical activity but increasing physical activity can lessen fatigue. Studies examining the acute effects of exercise on fatigue shed little insights on this issue, and findings vary based on intensity, whether fatigue symptoms are measured concomitantly or separately from feelings of energy, and the timing of the post-exercise measurement (Ensari, Sandroff, & Motl, 2016; Ensari, Sandroff, & Motl, 2017; Loy, O’Connor, & Dishman, 2013; Petruzzello & Motl, 2011). Therefore, it could be that perception of fatigue shortly after exercise presents as a perceived barrier to a behavior that, if completed regularly, could improve perception of fatigue severity in the long term.

A deeper investigation of the fatigue experience as it pertains to exercise and physical activity is imperative to improve the health of people with MS. A previous mixed methods investigation of fatigue in people with MS was conducted with the purpose to evaluate the fatigue experience in relation to emotional susceptibility and body sensations in people with MS (in-print). The parent investigation utilized quantitative survey data and qualitative interview data, both collected remotely. In the parent study, quantitative analyses focused on relationships between emotional susceptibility, interoceptive awareness, and fatigue. Inductive thematic analysis was utilized to describe the role of emotions and body sensations in the fatigue experience, and their collective impact on exercise. The parent investigation identified worry as a persistent issue related to the subjects’ thoughts and responses to exercise and physical activity, evidenced in interview data. Because of its pertinence, this discussion was pursued as a separate analysis, described below as a secondary analysis. The purpose of this study is to more completely investigate the role of worry related to fatigue in exercise and physical activity behavior in people with MS.

## Methods

### Study design

Mixed methods were chosen to assess the research question. The purpose of utilization of both methods is triangulation (Johnson & Onwuegbuzie, 2004). The present study sought to find convergence in qualitative data discussion of worry as it relates to the body and quantitative data assessment of interoceptive awareness. The typology utilized in this study was a sequential qualitatively-weighted design (Leech & Onwuegbuzie, 2009), with the sequential aspect of this design attributed to the parent study design. The quantitative data collection occurred first, followed by the qualitative data collection. The quantitative data and qualitative data were not weighted equally in interpretation of the findings for the present study; the qualitative data were weighted more heavily. This study utilized a single-group experimental design with all eligible participants undergoing the same survey collection and optional interview. The research question was addressed quantitatively in a cross-sectional manner. The survey consisted of demographic information collection as well as psychological primary outcome collection. The parent study for this study was approved by university-level IRB (ID: PROJECT00003248).

### Participant selection

Participants were recruited through various community and national advertisements and word-of-mouth techniques. Inclusion criteria were: a self-reported medical diagnosis of MS, being between the ages of 18 and 65, and identifying as being actively engaged in any physical activity (answering ‘yes’ to the question ‘Would you consider yourself to be physically active?’). Exclusion criteria included the absence of an MS diagnosis and no access to a reliable internet connection or phone for the survey and interview administration. If participants were deemed eligible, they moved forward to consent and data collection. Participants in this study were part of the previous study described above (in print).

### Data collection

#### Qualitative data collection

This study performs a secondary analysis of qualitative interview data, with the purpose of addressing a different research question (Heaton, 2008), which emerged during the previous analysis. Secondary analysis was a beneficial method to pursue in this context, as this interview data were collected from a relatively unique, difficult-to-access population on a topic that could be considered sensitive or emotional (Long-Sutehall, Sque, & Addington-Hall, 2010).

The methods of data collection were identical across the parent study and the current study. The interviews analyzed for this study took place after quantitative survey data collection. Interviews followed a semi-structured format (The SAGE, 2008), which allows for probing and clarifying questions while also following a topical guide. Interview questions were the same as those asked of participants for the companion study. Interview questions were targeted towards bodily sensations experienced with fatigue, perceptions of the meaning of the bodily sensations, emotional responses experienced with fatigue, perceptions of the meaning of the emotional responses, how these factors collectively affect activity choice in people with MS, how these factors shape confidence and efficacy in people with MS, and perspectives on the experience as a positive or negative feedback from the body (see supplement for interview guide). These interviews were designed to last one hour and were conducted over the phone. Audio recording was taken from these interviews. The audio recordings were used to create interview transcripts for analysis. Pseudonyms were provided for participants. Any other identifying information, such as names of family members, locations, etc. was substituted with a generic term (i.e. – ‘doctor’, ‘partner’, ‘nearby town’, ‘gym’). The first author performed all interviews and transcriptions. At the time of this data collection, the first author had taken several years of coursework in this area and led several qualitative inquiries leading to publication.

#### Quantitative data collection

The methods of data collection were identical across the parent study and the current study. Demographic information collected included age, gender, and race. Functional level was assessed using the Patient-Determined Disease Steps (PDDS) (Learmonth, Motl, Sandroff, Pula, & Cadavid, 2013) and habitual physical activity was assessed by the Godin Leisure-Time Exercise Questionnaire (GLTEQ) (Shephard, 1997).

Interoceptive awareness was chosen as a primary quantitative measure because of participant discussion of the worry; worry centered around body sensations and interpretation of sensations, so interoceptive awareness measurement could aid in understanding worries about body sensations. Interoceptive awareness was measured using the Multidimensional Assessment of Interoceptive Awareness-2 (MAIA-2) (Mehling, Acree, Stewart, Silas, & Jones, 2018). This is a 37-item scale, with 8 subscales. Subscales are: noticing (‘awareness of uncomfortable, comfortable, and neutral body sensations’), not-distracting (‘tendency not to ignore or distract oneself from sensations of pain or discomfort’), not-worrying (‘tendency not to worry or experience emotional distress with sensations of pain or discomfort’), attention regulation (‘ability to sustain and control attention to body sensations’), emotional awareness (‘awareness of the connection between body sensations and emotional states’), self-regulation (‘ability to regulate distress by attention to body sensations’), body listening (‘active listening to the body for insight’), and trust (‘experience of one’s body as safe and trustworthy’) (Mehling et al., 2018). Participants answer items on a Likert scale, grounded at 0 (‘never’) and 5 (‘always’). Subscale scores are calculated by taking the average score of items within the subscale. While this tool has not been validated in people with MS, it has been shown to have adequate psychometrics (Mehling et al., 2018).

### Data analysis

#### Qualitative data analysis

Qualitative data analysis was conducted after all interviews were transcribed and de-identified. The first author read over the transcripts multiple times to familiarize herself with the data. These data were analyzed utilizing constructivist grounded theory (Charmaz, 2000). Grounded theory itself is a qualitative research approach, ‘designed to support the inductive development of theory about a phenomenon through a set of systematic procedures for the collection and analysis of qualitative data’ (Locke, 2002). Constructivist grounded theory is different than traditional grounded theory in that it recognizes the role of the researcher in interpretation of meaning from theory variables and the focus becomes meaning, action, and process in a social situation. It also assumes the value of multiple realities existing in the context of a discussion of a phenomenon, taking into account the researcher and the participant’s point-of-view (Charmaz, 2000; Charmaz, 2006). The theory is developed and refined by an inductive, iterative coding process in which codes give rise to theory variables to help explain a process or social phenomenon (Hallberg, 2006). The resulting theory, centered around worry, is the primary outcome of qualitative data collected in this study. Reflexivity was considered during analysis and interpretation of meaning of interview data. The primary researcher did take individual reflexivity into account as it relates to emotional and relational ‘distance’ from the research participants (Mays & Pope, 2000; Meyrick, 2006), since some participants were involved in prior research. Analysis and transcription were completed by the first author with the use of analyzation software (Atlas.ti 9 for Windows).

#### Quantitative data analysis

Quantitative data analysis was performed using IBM SPSS Statistics 26. Scores of interoceptive awareness were calculated according to scale scoring instructions. The interoceptive awareness subscale scores (MAIA-2) were regressed in a multivariate linear regression model to assess potential relationships with habitual physical activity scores (GLETQ). A second multivariate linear regression model was created to assess the relationship of MAIA-2 subscale scores and GLETQ scores with control of PDDS scores.

## Results

Thirty-five participants completed the optional qualitative interview in addition to the quantitative survey collection. Fifty-five participants completed the quantitative survey collection only. Table 1 depicts participant demographic information for the 55 participants completing the survey collection.

**Table 1.** Participant characteristics (*n* = 55).

Characteristic	Mean (SD)/Percent
Age	48.1 years (11.7); Range: 24–64 years
Gender:	
*Male*	20.0%
*Female*	78.2%
*Other/Wish to not identify*	1.8%
Race/Ethnicity:	
*Asian*	0%
*Black/African*	5.5%
*Caucasian*	83.6%
*Hispanic/Latinx*	10.9%
*Native American*	0%
*Pacific Islander*	0%
*Other/Wish to not identify*	0%
Patient-Determined Disease Steps (PDDS) Score	2.1 (1.8); Range: 0–6
Godin Leisure-Time Exercise Questionnaire (GLETQ) Score	25.8 (22.4); Range: 0–91

### Qualitative results

The emerging theory describes participants’ thoughts and experiences regarding the consequences of fatigue during exercise, and how they influence subsequent actions. These thoughts and actions can be divided into respective parts of the overall theory. These parts do interact and have influence on each other, as is discussed in the subsequent sections.

#### Fatigue onset

Participants described the way that fatigue feels in several dimensions. They described body sensations associated with fatigue, and locations of those sensations. Many participants discussed heaviness as a sensation associated with fatigue. Heather described, ‘The heaviness, the heaviness is all over the body’. Kelly specified the location of heaviness: ‘ … the heaviness in the legs … ’. Participants also discussed pain as associated with fatigue. Leslie said:

… shooting pain … That would be normally in my lower back or in my hips. More if I, if my neck is sore then I’ll get that electrical shock you know when I bend my head down, whatever that’s called. Like electricity, I get that one when I am fatigued.

Frank described other sensations: ‘ … my feet start to burn like they’re on fire. Um, my hands, I get, I have the uh, the … pins and needles sensations in my hands.’ Amy was able to identify changes in heart rate: ‘ … when I get like really, really extremely fatigued, I do get um … shaky and … um, like rapid heart rates.’

Also, participants described emotions that arise when fatigue is present. While there were a diverse group of emotions reported, emotions centered around frustration. Charlene describes: ‘ … a lot of times it’s out of like frustration … I’m like you know, why do I have to feel like this, why does it have to be like this. It just pisses me off.’ Veronica said, ‘You know, like for me the frustration part happens when, first onset and you’re like oh no … wait, we are going down!’ Some participants also identified more ‘cool’ emotions as part of this experience. For example, Hilary said, ‘ … of course I, I’m very tired, but emotionally … I, I get very depressed um, there’s not so much anger as it is just … it, it’s more of a poor pitiful me kind of phase … ’. Zach said, ‘ … you know the big one is just feeling … uh, worthless I guess, useless … It’s impossible to not just kinda feel terrible about myself um when I’m fatigued like that.’

Together, these dimensions indicated to participants that they were beginning to feel fatigued. Participants described the awareness of fatigue as being tied to an awareness of specific body sensations, changes in function, and emotions. Renee described the process as,

so first you … you feel it, it’s like … uh, you know, and so your body sends you a very loud message, and then your brain acknowledges it and says ok I hear you, and then you, you know, it’s like you do like a little, what I would call a wellness check. You know, your brain, it’s like you’re checking your body and saying really? Really, you know is this what I’m feeling, and it’s like yeah … now that I’ve checked in with my body, it really is what I’m feeling … 

Kate discussed aspects of trust with her experience:

I guess I feel that my body knows things before my brain does? And I guess that’s how the thought process works, like the body has experiences that the mind then interprets … it’s just the, you know, the brain trying to listen to what the body sensations are saying and figure out … how much attention to pay to it.

The awareness also could change based on situation or personal choice to ignore the sensation and continue the activity. For example, Terri described a difference in situation as having an impact on perception of fatigue: ‘ … if I’m at home, I will be – I will listen to my body more and I will sit down and I will stop what I’m doing and when I feel better I will continue … ’.

#### Exercise specific worry and defensiveness

Participants described a worry that came from feelings of fatigue during exercise. The worry centered around body consequences of the fatigue. Several participants identified this as being related to relapse or worsening of the disease condition. For example, Jenny described her experience:

I do get cautious and scared before each workout … like is today gonna be the day I exacerbate it and I pass out and I have to go to the hospital … that could be me if I overdo it at the gym.

 Tara said: ‘ … because I’m always wondering, am I, am I going to pay for this? Meaning … if I do this … is it, is it going to make my fatigue worse or is it going to help?’ Rhonda explained: ‘ … when, when I get, you know fatigued and stuff, I’m thinking … oh no this is the time.’ Some participants discussed this worry in the context of fatigue causing an accident or injury while working out. Serena explained:

I don’t like to go out, doing any like hard core cardio if I’m feeling extra fatigued cause I don’t wanna make it worse … fear of um, all the sudden relapsing and falling while working out in front of other people.

Nicole said: ‘ … if I were to feel fatigued, I would … worry that I would be clumsy or fall or something like that while I’m doing any type of … like, exercise in that sense.’

Defensiveness was identified in the descriptions of participants as an avoidance of the fatigue, and a desire to return to a homeostatic state. For example, Kelly described her experience:

I kinda have in my mind ok, once my, once my body gets to feeling this way, then my mind knows ok let’s shut this down or scale it back. Cause you’re not gonna be able to finish, or, or you’re not gonna be able to do this safely.

Tina elaborated on her thoughts after getting fatigued:

Get someplace safe!  …  Not go out for a walk, not go out um, for a drive or anything like that, because I never wanna … I want to control the situation as much as I can. So it’s just, I sit down … so I become sedentary at that time until I feel better.

Miranda said:

… like going for a walk, like I would go for a walk and even when I do that, I’m kinda like ok … I think you’d better go the other direction now and go home! Cause you’re, you’re, you’re hitting your limit. And so even with that – and you can’t go beyond that … 

Zach discussed the maintenance of energy as part of defensiveness:

… energy is currency I guess you know? Like I have to have the energy to do these things. Um, so that means I can’t risk using it for these other things you know, going for the walk … you know, let alone like actually like ok, I’m gonna get a 30 minute workout in today. Um, it just seems … foolish, I guess. Um, and kind of like self-serving and self-defeating at the same time … 

#### Belief about benefits of physical activity

Although the worry from fatigue was a pervasive aspect of participants’ descriptions of the exercise experience, participants also identified benefits of consistently being physically active. Participants discussed these benefits as a rationale for engaging in physical activity. Some participants described physical benefits. Rita said, ‘I guess, just knowing that I can still (breathes) exercise and do things and know that those things are helping me to … feel stronger, and … keep me from getting, you know, weaker and more debilitated.’ Veronica added, ‘ … exercise is you know, just good for um … you know, general stamina. Which, you know, I need, I hope that I get that for sure, it helps my physical health.’. Some participants described mental or emotional benefits of being physically active. For example, Erica said, ‘ … if I’m, I’m feeling some kind of way, it helps, it helps me to go out and take a walk. It just helps me to clear my head and move around a little bit.’ Hannah added, ‘ … it’s the bit of respite I need to make it through the day if I exercise. And emotionally, I’m just … happier.’ Some participants even identified fatigue as an area that exercise improves. Natalie said,

… if I’m really fatigued, and I know that physical activity um can help me, uh, uh you know, persist through it um I will … um, do my best to try and engage in some sort of physical activity even if it’s minimum like stretching or just getting up and moving, walking around a little bit.

#### Belief in ability to do physical activity

Participants did hold fundamental beliefs about their ability to perform physical activity. However, those beliefs were subject to change when participants became fatigued during exercise. Participants discussed the fatigue – and resulting worry – as a hinderance to their belief. For example, Beverly said,

… it sorta hinders you because you start off with negativity I guess of how am I gonna start this when I can’t finish this. Or … because if you, it’s like if you start off tired doing something, and you know you’re gonna end up tireder … 

Hannah describes this: ‘I don’t say it out loud, but I’m like can I do it? Like, yeah, I mean I still do the stuff, I still do it, but … there is a little doubt there, in my ability to be … what I once was … ’. Natalie said: ‘ … it serves as a reminder um that I’m not as um physically able as I once was … It often, um, causes me to question my ability um, to uh perform those similar uh act, activities in the future.’ Trevor said:

Will I be able to last long enough to complete this task. And usually the thought is no … when it comes to things like exercises, because it’s so easy to overdo it and if I overdo it, I probably wont exercise for the next couple of days.

#### Exercise type, time, and frequency

Participants described their specific preferences regarding type of exercise, time spent exercising, and frequency of exercise. However, much like the belief in ability to do physical activity, this component of the model was also subject to change in a fatigued state. The changes could be made prior to start of exercise, or during exercise. These changes can be traced back to worry and defensiveness in exercise.

Participants expressed that fatigue could impact exercise type or mode chosen for a given day. Fatigue caused participants in this sample to avoid specific exercises. For example, Heather describes:

… there are some things I won’t do because of that … I avoid lifting things. You know, like I would lift, like if I would lift a weight, I wouldn’t do that. Um … and really that day I probably wouldn’t do anything with … raising your legs, like if you were to do up and down the stairs, I wouldn’t do that.

Amy explained,

… um it definitely impacts choices, because it’s that … you know, which one’s gonna make the most sense … the idea of like, having to get in the car and like find a thing, and do the hike, do the thing, and like go hiking – which I would love to do, it’s just, that’s not … a feasible choice as much anymore, especially because I’m like well what if I like get knocked out like halfway through the thing, and then I gotta sit there for a while before I can get back.

Jenny describes her experience: ‘ … sometimes I do have to pull back and stop or do yoga instead … so like if I’m more fatigued and my body just isn’t feeling right I’ll do yoga … ’

Exercise time could also be influenced by fatigue. For many participants, fatigue meant that exercise time had to be cut short. Heather explained:

… depending on how bad it is, I’ll go a couple of miles, if I’m having a really, really good day. Um normal day, I … I kinda go a few houses down and that’s as far as I can make it. Terrible day, I can go to the end of my driveway … and that, I have a pretty, a long driveway.

Valerie said: ‘if I’m tired, I can’t, I can’t keep going. So I, let’s say I’m on a walk and … if I’m tired, then I have to turn around and then I will perhaps be upset … ’. Fatigue also had an impact on how much time was budgeted for exercise up front. For example, Frank said,

… you know, it’s two hours that, that two hours I know it’s, it’s what I can do. Um … uh, you know, whether it’s yoga or stretching or biking or, or lifting or … you know, working, working on my legs. I, I’ve noticed there is a certain amount of time of physical activity [exercise] that I’m gonna shut down.

Adam added,

There are times when I can go – you know, you can do 20 min on a bike and that’s 4 miles or … so you know when I get done, you know my legs are tired and I expect them to be … 

Also, time of day for exercise was impacted by fatigue. For example, Veronica described how weather and time of day impacted fatigue: ‘ … we were walking 4 miles a day and that’s not a cause of fatigue unless it’s super hot. Um, and that’s about choosing the right time of day … ’

Participants described the effect of fatigue on exercise frequency. Miranda described this as: ‘ … you don’t, you don’t do much … that’s not gonna happen if I have physical problems. Uh, like uh fatigue. But, your physical activity [exercise] goes way down, you don’t really move a lot … ’ Veronica said, ‘ … so comparing that to running or strength training or things like that that are more likely to cause fatigue, um, yeah, they’re … I’m you know, to a point where it could impact frequency.’ Amy described her experience:

… because when I’m not feeling as much fatigue, I exercise more … it’s like ok I can do like the one big thing … once a week. Like I can go for like a big, long walk or … a little bit more of like vigorous activity … 

#### Narrative description of theory model

**Figure 1.**
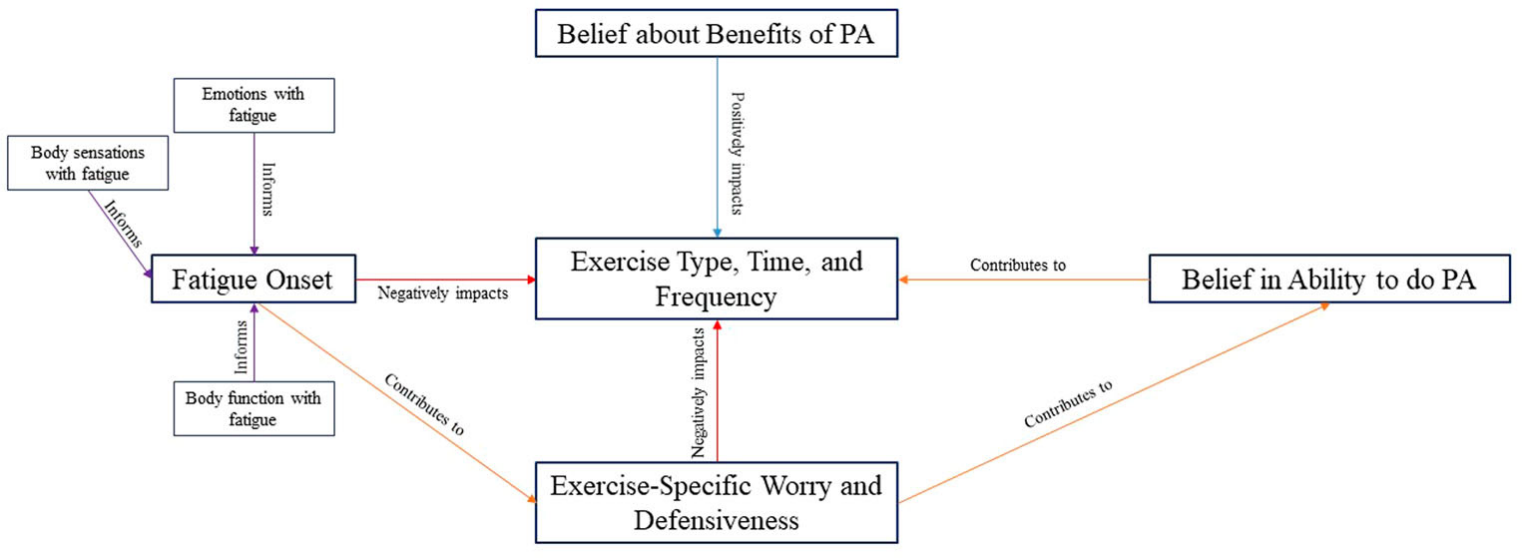
Grounded theory model results.

As people with MS start to fatigue during exercise, they begin to experience worry and defensiveness associated with the body sensations, emotions, and changes in function that fatigue causes. These fatigue indicators cause participants to worry about the status of their body and changes that severe fatigue could cause to that status. They also worry about declines in function that could lead to an injury or fall during exercise. This worry and defensiveness coexist with beliefs about benefits of physical activity; alterations in exercise time, type, and frequency of exercise are not the result of a lack of knowledge about physical activity benefit. Rather, they are the result of the impact of worry and defensiveness in exercise. The worry and defensiveness can further change perspectives on ability to complete an exercise or physical activity. This is evidenced by participants’ discussions of feelings of being unable to complete an exercise in the moment as well as discussion of what they believe themselves to be capable of after an incident with worry. The worry, defensiveness, and their effects on belief to perform exercise or physical activity all contribute to decisions about exercise time, type, and frequency. Figure 1 displays the components of the model and their interactions.

### Quantitative results

MAIA-2 subscale scores weakly predicted Godin scores ($R^{2}$_ _= 0.19). With the addition of PDDS into the model with MAIA-2 subscale scores, fit improved ($R^{2}$_ _= 0.27), thus 27% of the data fit the model (Table 2).

**Table 2.** Regression analysis results.

Model	R	$R^{2}$	Standard error of the estimate	$R^{2}$ Change	F Change	Sig. F Change
1^a^	0.43	0.19	21.93	0.19	1.32	0.26
2^b^	0.52	0.27	21.02	0.08	5.08	0.03

^a^ Predictors: (Constant), Trusting, Noticing, Notdistracting, Selfregulation, Emotionalawareness, Bodylistening, Attentionregulation, Notworrying.

^b^ Predictors: (Constant), Trusting, Noticing, Notdistracting, Selfregulation, Emotionalawareness, Bodylistening, Attentionregulation, Notworrying, PDDS.

There were statistical differences in individual coefficients within the regression model. Before the addition of PDDS, the ‘Not worrying’ subscale score was a statistically significant predictor of Godin scores (β = −0.40, *p* =  0.05) (Table 3). However, once controlling for PDDS, ‘Not worrying’ is no longer a significant predictor of Godin scores; rather, PDDS becomes a statistically significant predictor (β =  −0.33, *p* =  0.03) (Table 3).

**Table 3.** Regression models 1 and 2 coefficients table (*n* = 55).

Model 1					Model 2			
Effect	Estimate (B)	SE	Standardized Beta (β)	*P*-value	Estimate (B)	SE	Standardized Beta (β)	*P*-value
Intercept	40.71	24.24		0.10	55.67	24.16		0.02
Noticing	−5.72	4.87	−0.20	0.25	−5.98	4.67	−0.21	0.21
Not Distracting	2.97	3.72	0.13	0.43	2.44	3.58	0.11	0.50
Not Worrying	−8.68	4.27	−0.40	0.05*	−7.16	4.16	−0.33	0.09
Attention Regulation	3.60	4.21	0.16	0.40	5.85	4.16	0.26	0.17
Emotional Awareness	−0.93	4.04	−0.04	0.82	−0.81	3.87	−0.04	0.84
Self-Regulation	6.06	5.61	0.23	0.29	3.40	5.51	0.13	0.55
Body Listening	−0.70	3.34	−0.04	0.84	−1.45	3.22	−0.08	0.66
Trusting	2.41	4.60	0.09	0.84	0.16	4.52	0.01	0.97
Patient-Determined Disease Steps		−4.19	1.86	−0.33	0.03*			

*value significant at alpha = 0.05 level.

### Mixed methods results

Qualitatively, participants discussed how fatigue caused body-centered worry, especially around potential accidents during exercise or changes in body status because of the fatigue. This body-centered worry negatively impacted exercise behavior, causing alterations in type, time, and frequency. This worry also impacted belief in ability to do exercise. This occurred not only with specific exercise types, but also with specific durations. Discussion indicated that less physical activity is taking place when fatigued, and worry had an intricate role to play in the decision to be less physically active when fatigued.

The quantitative assessment of subscale scores related to aspects of interoceptive awareness regressed on habitual physical activity scores and showed that the ‘Not worrying’ subscale of the MAIA-2 is a predictor of physical activity. The ‘Not worrying’ subscale scores refer to the, ‘tendency not to worry or experience emotional distress with sensations of pain or discomfort.’ (Mehling et al., 2018) This sample showed that those with lower physical activity scores tended to have higher not worrying scores. However, with the addition of PDDS into the regression model, ‘Not worrying’ was no longer a statistically significant predictor of habitual physical activity levels. Rather, function became the only statistically significant predictor. This finding does contradict the finding about the role of worry qualitatively – that increased worry drives physical activity levels down.

In summary, there was dissonance between qualitative discussion of the negative impact of worry on exercise and quantitative evaluation of aspects of interoceptive awareness and exercise. The relationship between body-centered worry and body sensation awareness (interoceptive awareness) does not seem to be clearer from this investigation.

## Discussion

Worry around exercise was an important part of the discussion of the fatigue experience in people with MS. Qualitatively, worry has been examined in several contexts in the MS literature. For example, worry has been examined in association with the period surrounding the initial diagnosis of MS (Koopman & Schweitzer, 1999). Worry has also been discussed in the context of MS relapse (Matza et al., 2019). However, worry in the context of physical activity is less documented. In an examination of the lived experience of individuals with MS with exercise, worry about individual safety with exercise equipment was briefly documented (Borkoles, Nicholls, Bell, Butterly, & Polman, 2008). In an examination of facilitators and barriers to physical activity engagement in people with MS, worry was briefly discussed in the context of fatigue during exercise (Kayes, McPherson, Taylor, Schluter, & Kolt, 2011b). This discussion does corroborate several of the concepts discussed by participants in this sample. For example, Kayes et al. gives a sample quote to demonstrate fatigue as a barrier related to management:

Mainly, it’s the worry about the fatigue, if I overdo it, I get the fatigue and you know, sort of balancing the two … I balance my life around it, you know. We had a party Saturday night … I rested most of Saturday afternoon, so that I could enjoy the evening. (Hannah,, age 39). (Kayes, McPherson, Taylor, Schluter, & Kolt, 2011b)

This kind of worry that comes with fatigue was discussed by our sample, although perhaps not as frequently as other aspects of worry. The results of the current study illuminate and contextualize worry around exercise fatigue in a novel way.

A separate, quantitative investigation of facilitators and barriers by Kayes et al. (2011) is more closely related to the results of this study (Kayes, McPherson, Schluter, Taylor, Leete, & Kolt, 2011a). This investigation involved administration of fear-avoidance measurement tools focused on symptom responses (Moss-Morris & Chalder, 2003). Some items on this tool are specific to exercise; for example, ‘I am afraid that I will make my symptoms worse if I exercise’ (Moss-Morris & Chalder, 2003). However, analysis of data by Kayes et al. revealed that fear avoidance of exercise was more related to risk of injury than symptom worsening (Kayes, McPherson, Schluter, Taylor, Leete, & Kolt, 2011a). This does corroborate aspects of the qualitative data collected in the current sample. Discussion of body-related consequences of exercise did include fall risk increasing with fatigue during exercise. However, the amount of discussion related to symptom worsening or relapse with fatigue in exercise was more prevalent in the current sample. This could be because of the orientation of the qualitative interview towards perception of body sensations, as to capture interoceptive awareness aspects. What these data do suggest is that there could be a symptom-exacerbation specific worry that was captured qualitatively but not captured quantitatively. While this could be because of aspects of survey-related bias or comfortability discussing these topics rather than answering survey items, it does warrant further investigation of perspectives of people with MS.

The findings of this study indicate that aspects of interoceptive awareness are not necessarily accurate predictors of physical activity in people with MS. While this could be because of lack of validation of the MAIA-2 among people with MS, there is evidence to suggest that the MAIA-2 might not have a desirable sensitivity to all aspects of interoceptive awareness or sensibility (Desmedt, Heeren, Corneille, & Luminet, 2021). A review that compares current self-report interoception scales does show that the MAIA-2 could be best used to measure tendency not to worry about internal sensations (‘Not worrying’ subscale) compared to other interoceptive awareness scales, but perhaps not best for other aspects of interoceptive awareness (Desmedt et al., 2021). While this observation supports the conclusion of the role of worry in this analysis, it does also suggest that other aspects of this analysis could be less accurate and not representative of the role of the subscale constructs. This sample in particular averaged higher than healthy individuals on the ‘Not worrying’ subscale (3.0 vs. 2.3, respectively, Ferentzi et al., 2020).

Theoretically, the finding that PDDS predicts physical activity is sound, since functionality has been associated with physical activity in people with MS (Kayes, McPherson, Schluter, Taylor, Leete, & Kolt, 2011a; Streber, Peters, & Pfeifer, 2016). Generalized worry has also been linked to disability status in people with MS, with increasing disability related to increased generalized worry (Bruce & Arnett, 2009). It is important to note that generalized worry was not quantitatively assessed in this sample. It could be, then, that those with increasing disability status do not engage in physical activity as often or as strenuously, due to generalized worry pertaining to their body status, as hypothesized by Bruce and Arnett (2009) (Bruce & Arnett, 2009). While this sentiment agrees with qualitative discussion of body-related worry about the consequences of fatigue, further analysis of the quantitative data from this sample indicates no collinearity between model variables, including PDDS and the ‘not worrying’ subscale. This would suggest that these constructs are not related quantitatively in this sample, as seen in Bruce and Arnett (2009). Worry was measured differently in both studies; worry was a secondary consequence of body awareness in this study, while Bruce and Arnett (2009) measured worry with the Pennsylvania State Worry Questionnaire (PSWQ) (Bruce & Arnett, 2009; Molina & Borkovec, 1994). The specificity of these tools could offer an explanation to the dissonance.

The dissonance seen in qualitative discussion of worry surrounding the body during exercise and quantitative aspects of interoceptive awareness could have come from a variety of sources. Discussion of physical activity in the qualitative interviews was centered more on the acute effect of worry and fatigue; scenarios commonly discussed included thoughts and actions when fatigue begins. The MAIA-2 is designed to measure trait interoceptive awareness, meaning that a subscale score is meant to be interpreted as a score that indexes individuals’ relatively permanent dispositions. Therefore, the results of this study indicate worry could have a stronger negative effect acutely than chronically; this would indicate that worry chronically could push individuals with MS into more health-protective behaviors. Evidence of this does exist in the qualitative discussion of the diagnosis experience in people with MS (Koopman & Schweitzer, 1999). A more specific exploration of worry and health behavior in the MS population is warranted.

As previously mentioned, worry is part of a spectrum of anxiety-depressive symptoms in people with MS. It is important to note that these symptoms come, in part, from the unpredictable nature of the disease (Desborough et al., 2020). The most common type of MS, relapsing-remitting MS (RRMS), is characterized by unpredictable relapses which result in progression of the disease and loss of function, which could be a significant contributor to one’s perception of body sensation and worry. One qualitative study of the phenomenon of an RRMS diagnosis described participants’ experiences as a balancing act, with one participant stating,

Everything that happens to you is like a learning process that you have to go through. It gets hard when symptoms occur again. When it’s kind of stable you can put it to the background giving you the feeling that nothing is going on. But then you encounter new complaints, things that you’ve never had before and that scares me …  (Hvd, Dv, van der Hiele, & Visser, 2018)

A qualitative investigation of perceptions of safety in exercise during a MS relapse in RRMS participants found that relapses do disrupt normal physical activity schedules, making acute exercise difficult to adhere to (Wilkinson, McGraw, Chung, & Kyratsis, 2022). Further studies should investigate the role of worry in individuals with RRMS, as the effect of the relapses on health behaviors like physical activity might be significantly different than observed in other types of MS.

While this work does point to the potential issue of worry in people with MS, the scope does not extend to treatment or addressing of worry. In psychological research and practice, exposure therapy is often utilized to combat feelings of worry or anxiety (Follette & Smith, 2005; van der Heiden & ten Broeke, 2009). Exposure therapy treatment involves exposing an individual to a stimulus to break the pattern of fear and avoidance of the desired target (Follette & Smith, 2005). These results do relate to the work of exposure therapy research in that worry, in this context, could be tied to exposure to fatigue or physical activity in this sample. Mindfulness training has also demonstrated benefit in people with MS, particularly by encouraging self-acceptance of the presence of emotions like worry (Burschka et al., 2014; Sauder et al., 2021). Combinations of exercise interventions and mindfulness interventions that encourage acceptance of worry could be tailored to help overcome worry and long-term physical activity or exercise behavior. Further research along these lines could test the usefulness of exposure therapy or mindfulness for fatigue-related worry in people with MS.

### Limitations

This study is not without limitations. Firstly, this study does have a smaller sample size with regard to the quantitative data collection, which brings those results under scrutiny for sample size bias. However, qualitatively, an adequate sample size was obtained for the purposes of grounded theory. This sample is also quite functional with an average PDDS score of 2.1. Therefore, the quantitative results might not be applicable to those who are less functional or outside the exclusion criteria; similarly, this qualitative depiction of the experience could be different for people with MS who are less functional than this sample. Further work in this area should be done to determine the role of worry in people with MS who are less functional. Secondly, direct measures of anxiety and depression were not administered as part of this study and could have given more insight into the sample’s disposition. Clinical measures, such as those related to anxiety or depression, type of MS, and precise measures of physical functionality (i.e. – EDSS) were not obtained as part of this study and could have been useful in further characterizing the sample and interpretation of results. The analysis of the qualitative data was performed without the support of a secondary reviewer or auditor, which is a limitation. Finally, these data were collected during the COVID-19 pandemic, which could have an impact on perspectives of worry or anxiety in general, not just surrounding physical activity. Remote administration of interview due to COVID-19 could also have had implications on the recognizability of certain constructs in interviews or recognition of certain nonverbal communication constructs.

## Conclusions

This study found that people with MS who report being physical active present with strong qualitative themes related to worry about the impact and consequences of being physically active. This worry did not always cause cessation in physical activity, but it does represent a potential barrier to physical activity not commonly discussed in the MS literature. Further studies should examine worry surrounding being physically active in a deeper fashion. In addition, efforts to recommend and increase physical activity behavior in people with MS need to uncover and address worry related to being physically active.

## Supplementary Material

Supplemental Material

## Data Availability

The data that support the findings of this study are available from the corresponding author upon request.
