# Supplementary material for: The role of worry in exercise and physical activity behavior of people with multiple sclerosis
Source: Health Psychol Behav Med. 2022 Aug 18;10(1):786–805. doi: 10.1080/21642850.2022.2112197 (PMC9397454; doi:10.1080/21642850.2022.2112197)
Supplement: Supplemental Material [file RHPB_A_2112197_SM5371.docx]

**Interview Guide**

1. Can you describe what it feels like to be fatigued?
   1. What does it feel like in your body?
   2. What does it feel like emotionally?
2. Can you describe the sensations in your body when you are fatigued?
   1. What do the sensations in your body tell you?
   2. How do you process your body sensations when you are fatigued?
   3. Do you ever feel that your body sensations do not match your thoughts about what you're feeling? If so, how?
3. Can you describe the emotions you feel when you are fatigued?
   1. What do the emotions you feel when you are fatigued tell you?
   2. How do you process the emotions with fatigue?
   3. Do you feel that your emotions impact the way you perceive your fatigue? If so, how?
4. Do you think these things- fatigue, your body sensations, and emotions- impact your physical activity?
   1. Can you describe the ways that these things impact your physical activity?
   2. Do these things impact physical activity choices?
   3. Do these things impact physical activity frequency?
5. Do these things- fatigue, your body sensations, and emotions- impact your belief in your ability to perform physical activity?
   1. If so, how?
   2. Does the impact affect your belief each day or over many days?
6. Overall, when you hear the word fatigue, what do you think of?
   1. Does the word fatigue carry a connotation to you? If so, what is it?
   2. Do you feel that this connotation affects the emotions that you feel with fatigue? If so, how?
7. What do you think fatigue means for your overall functioning?
